# Supplementary material for: The Isocaloric Substitution of Plant-Based and Animal-Based Protein in Relation to Aging-Related Health Outcomes: A Systematic Review
Source: Nutrients. 2022 Jan 9;14(2):272. doi: 10.3390/nu14020272 (PMC8781188; doi:10.3390/nu14020272)
Supplement: Supplementary file 1 [file nutrients-14-00272-s001.zip › nutrients-1488709-supplementary.pdf]

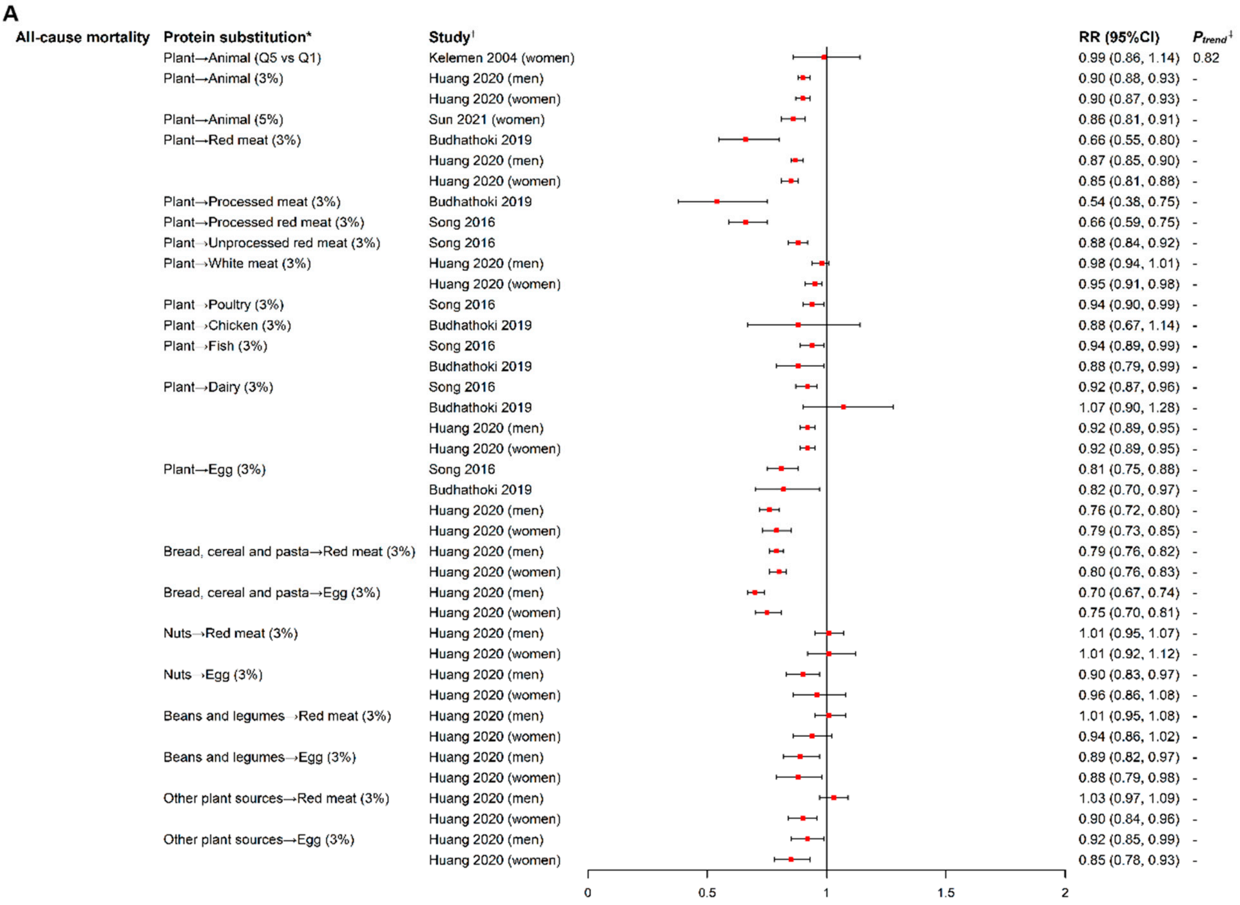
A
All-cause mortality
Protein substitution*
Study†
RR (95%CI)
P_{trend}‡
Plant→Animal (Q5 vs Q1) | Kelemen 2004 (women) | 0.99 (0.86, 1.14) | 0.82
Plant→Animal (3%) | Huang 2020 (men) | 0.90 (0.88, 0.93) | -
Huang 2020 (women) | 0.90 (0.87, 0.93) | -
Plant→Animal (5%) | Sun 2021 (women) | 0.86 (0.81, 0.91) | -
Plant→Red meat (3%) | Budhathoki 2019 | 0.66 (0.55, 0.80) | -
Huang 2020 (men) | 0.87 (0.85, 0.90) | -
Huang 2020 (women) | 0.85 (0.81, 0.88) | -
Plant→Processed meat (3%) | Budhathoki 2019 | 0.54 (0.38, 0.75) | -
Plant→Processed red meat (3%) | Song 2016 | 0.66 (0.59, 0.75) | -
Plant→Unprocessed red meat (3%) | Song 2016 | 0.88 (0.84, 0.92) | -
Plant→White meat (3%) | Huang 2020 (men) | 0.98 (0.94, 1.01) | -
Huang 2020 (women) | 0.95 (0.91, 0.98) | -
Plant→Poultry (3%) | Song 2016 | 0.94 (0.90, 0.99) | -
Plant→Chicken (3%) | Budhathoki 2019 | 0.88 (0.67, 1.14) | -
Plant→Fish (3%) | Song 2016 | 0.94 (0.89, 0.99) | -
Budhathoki 2019 | 0.88 (0.79, 0.99) | -
Plant→Dairy (3%) | Song 2016 | 0.92 (0.87, 0.96) | -
Budhathoki 2019 | 1.07 (0.90, 1.28) | -
Huang 2020 (men) | 0.92 (0.89, 0.95) | -
Huang 2020 (women) | 0.92 (0.89, 0.95) | -
Plant→Egg (3%) | Song 2016 | 0.81 (0.75, 0.88) | -
Budhathoki 2019 | 0.82 (0.70, 0.97) | -
Huang 2020 (men) | 0.76 (0.72, 0.80) | -
Huang 2020 (women) | 0.79 (0.73, 0.85) | -
Bread, cereal and pasta→Red meat (3%) | Huang 2020 (men) | 0.79 (0.76, 0.82) | -
Huang 2020 (women) | 0.80 (0.76, 0.83) | -
Bread, cereal and pasta→Egg (3%) | Huang 2020 (men) | 0.70 (0.67, 0.74) | -
Huang 2020 (women) | 0.75 (0.70, 0.81) | -
Nuts→Red meat (3%) | Huang 2020 (men) | 1.01 (0.95, 1.07) | -
Huang 2020 (women) | 1.01 (0.92, 1.12) | -
Nuts→Egg (3%) | Huang 2020 (men) | 0.90 (0.83, 0.97) | -
Huang 2020 (women) | 0.96 (0.86, 1.08) | -
Beans and legumes→Red meat (3%) | Huang 2020 (men) | 1.01 (0.95, 1.08) | -
Huang 2020 (women) | 0.94 (0.86, 1.02) | -
Beans and legumes→Egg (3%) | Huang 2020 (men) | 0.89 (0.82, 0.97) | -
Huang 2020 (women) | 0.88 (0.79, 0.98) | -
Other plant sources→Red meat (3%) | Huang 2020 (men) | 1.03 (0.97, 1.09) | -
Huang 2020 (women) | 0.90 (0.84, 0.96) | -
Other plant sources→Egg (3%) | Huang 2020 (men) | 0.92 (0.85, 0.99) | -
Huang 2020 (women) | 0.85 (0.78, 0.93) | -
0
0.5
1
1.5
2

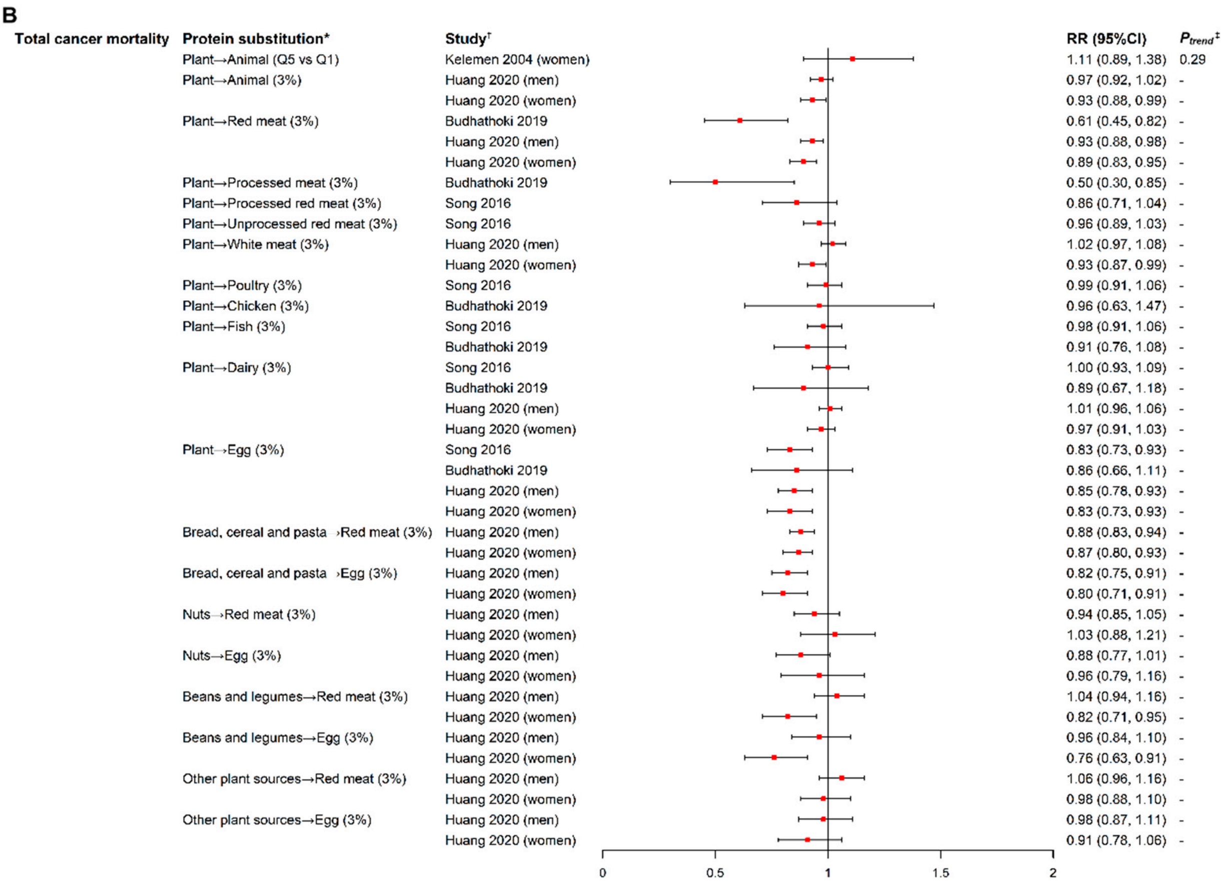
B
Total cancer mortality
Protein substitution*
Study†
RR (95%CI)
P_{trend}‡
Plant→Animal (Q5 vs Q1) | Kelemen 2004 (women) | 1.11 (0.89, 1.38) | 0.29
Plant→Animal (3%) | Huang 2020 (men) | 0.97 (0.92, 1.02) | -
Huang 2020 (women) | 0.93 (0.88, 0.99) | -
Plant→Red meat (3%) | Budhathoki 2019 | 0.61 (0.45, 0.82) | -
Huang 2020 (men) | 0.93 (0.88, 0.98) | -
Huang 2020 (women) | 0.89 (0.83, 0.95) | -
Plant→Processed meat (3%) | Budhathoki 2019 | 0.50 (0.30, 0.85) | -
Plant→Processed red meat (3%) | Song 2016 | 0.86 (0.71, 1.04) | -
Plant→Unprocessed red meat (3%) | Song 2016 | 0.96 (0.89, 1.03) | -
Plant→White meat (3%) | Huang 2020 (men) | 1.02 (0.97, 1.08) | -
Huang 2020 (women) | 0.93 (0.87, 0.99) | -
Plant→Poultry (3%) | Song 2016 | 0.99 (0.91, 1.06) | -
Plant→Chicken (3%) | Budhathoki 2019 | 0.96 (0.63, 1.47) | -
Plant→Fish (3%) | Song 2016 | 0.98 (0.91, 1.06) | -
Budhathoki 2019 | 0.91 (0.76, 1.08) | -
Plant→Dairy (3%) | Song 2016 | 1.00 (0.93, 1.09) | -
Budhathoki 2019 | 0.89 (0.67, 1.18) | -
Huang 2020 (men) | 1.01 (0.96, 1.06) | -
Huang 2020 (women) | 0.97 (0.91, 1.03) | -
Plant→Egg (3%) | Song 2016 | 0.83 (0.73, 0.93) | -
Budhathoki 2019 | 0.86 (0.66, 1.11) | -
Huang 2020 (men) | 0.85 (0.78, 0.93) | -
Huang 2020 (women) | 0.83 (0.73, 0.93) | -
Bread, cereal and pasta→Red meat (3%) | Huang 2020 (men) | 0.88 (0.83, 0.94) | -
Huang 2020 (women) | 0.87 (0.80, 0.93) | -
Bread, cereal and pasta→Egg (3%) | Huang 2020 (men) | 0.82 (0.75, 0.91) | -
Huang 2020 (women) | 0.80 (0.71, 0.91) | -
Nuts→Red meat (3%) | Huang 2020 (men) | 0.94 (0.85, 1.05) | -
Huang 2020 (women) | 1.03 (0.88, 1.21) | -
Nuts→Egg (3%) | Huang 2020 (men) | 0.88 (0.77, 1.01) | -
Huang 2020 (women) | 0.96 (0.79, 1.16) | -
Beans and legumes→Red meat (3%) | Huang 2020 (men) | 1.04 (0.94, 1.16) | -
Huang 2020 (women) | 0.82 (0.71, 0.95) | -
Beans and legumes→Egg (3%) | Huang 2020 (men) | 0.96 (0.84, 1.10) | -
Huang 2020 (women) | 0.76 (0.63, 0.91) | -
Other plant sources→Red meat (3%) | Huang 2020 (men) | 1.06 (0.96, 1.16) | -
Huang 2020 (women) | 0.98 (0.88, 1.10) | -
Other plant sources→Egg (3%) | Huang 2020 (men) | 0.98 (0.87, 1.11) | -
Huang 2020 (women) | 0.91 (0.78, 1.06) | -
0
0.5
1
1.5
2

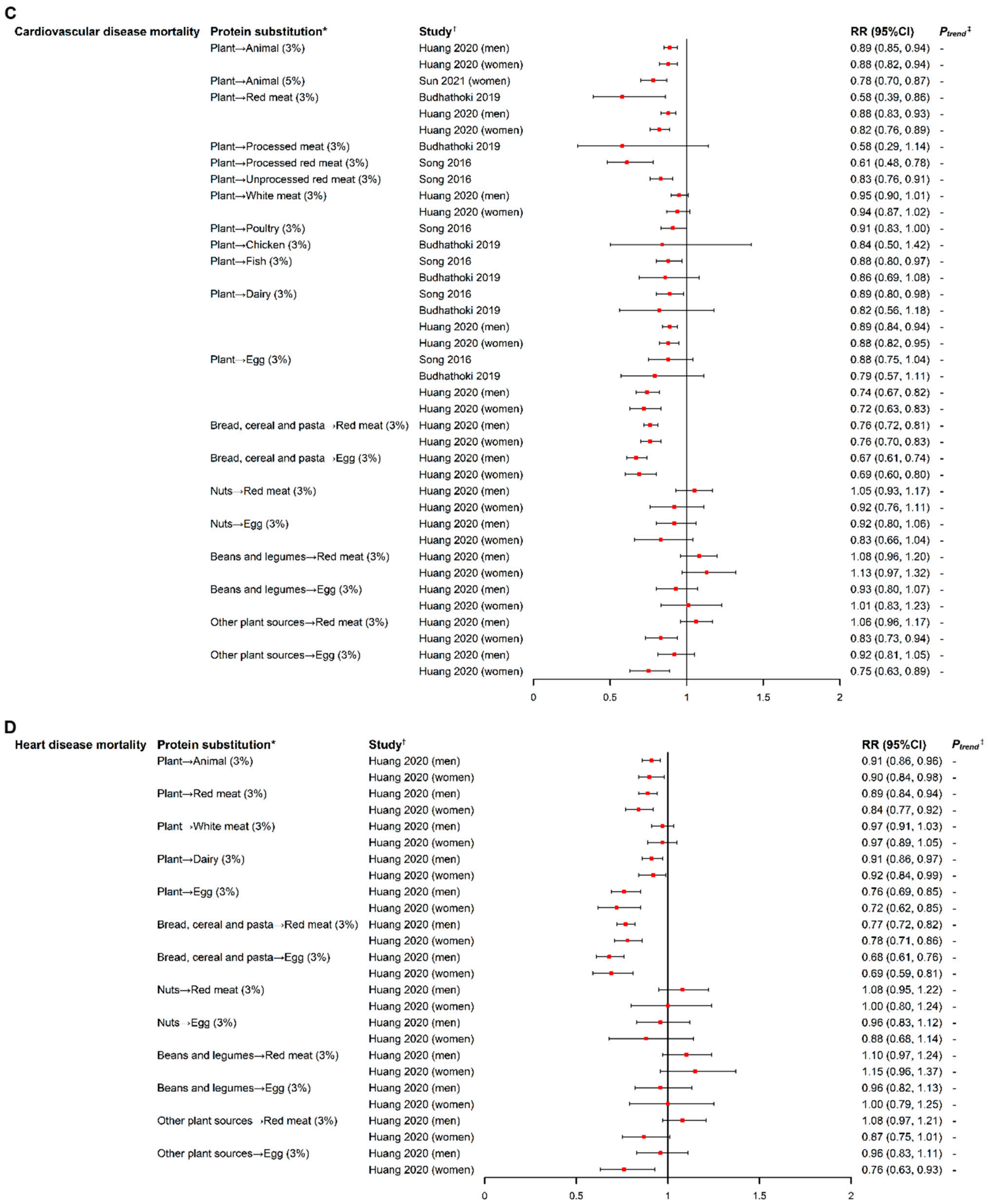
C
Cardiovascular disease mortality
Protein substitution*
Study†
RR (95%CI)
P_{trend}‡
Plant→Animal (3%) | Huang 2020 (men) | 0.89 (0.85, 0.94) | -
Huang 2020 (women) | 0.88 (0.82, 0.94) | -
Plant→Animal (5%) | Sun 2021 (women) | 0.78 (0.70, 0.87) | -
Plant→Red meat (3%) | Budhathoki 2019 | 0.58 (0.39, 0.86) | -
Huang 2020 (men) | 0.88 (0.83, 0.93) | -
Huang 2020 (women) | 0.82 (0.76, 0.89) | -
Plant→Processed meat (3%) | Budhathoki 2019 | 0.58 (0.29, 1.14) | -
Plant→Processed red meat (3%) | Song 2016 | 0.61 (0.48, 0.78) | -
Plant→Unprocessed red meat (3%) | Song 2016 | 0.83 (0.76, 0.91) | -
Plant→White meat (3%) | Huang 2020 (men) | 0.95 (0.90, 1.01) | -
Huang 2020 (women) | 0.94 (0.87, 1.02) | -
Plant→Poultry (3%) | Song 2016 | 0.91 (0.83, 1.00) | -
Plant→Chicken (3%) | Budhathoki 2019 | 0.84 (0.50, 1.42) | -
Plant→Fish (3%) | Song 2016 | 0.88 (0.80, 0.97) | -
Budhathoki 2019 | 0.86 (0.69, 1.08) | -
Plant→Dairy (3%) | Song 2016 | 0.89 (0.80, 0.98) | -
Budhathoki 2019 | 0.82 (0.56, 1.18) | -
Huang 2020 (men) | 0.89 (0.84, 0.94) | -
Huang 2020 (women) | 0.88 (0.82, 0.95) | -
Plant→Egg (3%) | Song 2016 | 0.88 (0.75, 1.04) | -
Budhathoki 2019 | 0.79 (0.57, 1.11) | -
Huang 2020 (men) | 0.74 (0.67, 0.82) | -
Huang 2020 (women) | 0.72 (0.63, 0.83) | -
Bread, cereal and pasta→Red meat (3%) | Huang 2020 (men) | 0.76 (0.72, 0.81) | -
Huang 2020 (women) | 0.76 (0.70, 0.83) | -
Bread, cereal and pasta→Egg (3%) | Huang 2020 (men) | 0.67 (0.61, 0.74) | -
Huang 2020 (women) | 0.69 (0.60, 0.80) | -
Nuts→Red meat (3%) | Huang 2020 (men) | 1.05 (0.93, 1.17) | -
Huang 2020 (women) | 0.92 (0.76, 1.11) | -
Nuts→Egg (3%) | Huang 2020 (men) | 0.92 (0.80, 1.06) | -
Huang 2020 (women) | 0.83 (0.66, 1.04) | -
Beans and legumes→Red meat (3%) | Huang 2020 (men) | 1.08 (0.96, 1.20) | -
Huang 2020 (women) | 1.13 (0.97, 1.32) | -
Beans and legumes→Egg (3%) | Huang 2020 (men) | 0.93 (0.80, 1.07) | -
Huang 2020 (women) | 1.01 (0.83, 1.23) | -
Other plant sources→Red meat (3%) | Huang 2020 (men) | 1.06 (0.96, 1.17) | -
Huang 2020 (women) | 0.83 (0.73, 0.94) | -
Other plant sources→Egg (3%) | Huang 2020 (men) | 0.92 (0.81, 1.05) | -
Huang 2020 (women) | 0.75 (0.63, 0.89) | -
0
0.5
1
1.5
2
D
Heart disease mortality
Protein substitution*
Study†
RR (95%CI)
P_{trend}‡
Plant→Animal (3%) | Huang 2020 (men) | 0.91 (0.86, 0.96) | -
Huang 2020 (women) | 0.90 (0.84, 0.98) | -
Plant→Red meat (3%) | Huang 2020 (men) | 0.89 (0.84, 0.94) | -
Huang 2020 (women) | 0.84 (0.77, 0.92) | -
Plant→White meat (3%) | Huang 2020 (men) | 0.97 (0.91, 1.03) | -
Huang 2020 (women) | 0.97 (0.89, 1.05) | -
Plant→Dairy (3%) | Huang 2020 (men) | 0.91 (0.86, 0.97) | -
Huang 2020 (women) | 0.92 (0.84, 0.99) | -
Plant→Egg (3%) | Huang 2020 (men) | 0.76 (0.69, 0.85) | -
Huang 2020 (women) | 0.72 (0.62, 0.85) | -
Bread, cereal and pasta→Red meat (3%) | Huang 2020 (men) | 0.77 (0.72, 0.82) | -
Huang 2020 (women) | 0.78 (0.71, 0.86) | -
Bread, cereal and pasta→Egg (3%) | Huang 2020 (men) | 0.68 (0.61, 0.76) | -
Huang 2020 (women) | 0.69 (0.59, 0.81) | -
Nuts→Red meat (3%) | Huang 2020 (men) | 1.08 (0.95, 1.22) | -
Huang 2020 (women) | 1.00 (0.80, 1.24) | -
Nuts→Egg (3%) | Huang 2020 (men) | 0.96 (0.83, 1.12) | -
Huang 2020 (women) | 0.88 (0.68, 1.14) | -
Beans and legumes→Red meat (3%) | Huang 2020 (men) | 1.10 (0.97, 1.24) | -
Huang 2020 (women) | 1.15 (0.96, 1.37) | -
Beans and legumes→Egg (3%) | Huang 2020 (men) | 0.96 (0.82, 1.13) | -
Huang 2020 (women) | 1.00 (0.79, 1.25) | -
Other plant sources→Red meat (3%) | Huang 2020 (men) | 1.08 (0.97, 1.21) | -
Huang 2020 (women) | 0.87 (0.75, 1.01) | -
Other plant sources→Egg (3%) | Huang 2020 (men) | 0.96 (0.83, 1.11) | -
Huang 2020 (women) | 0.76 (0.63, 0.93) | -
0
0.5
1
1.5
2

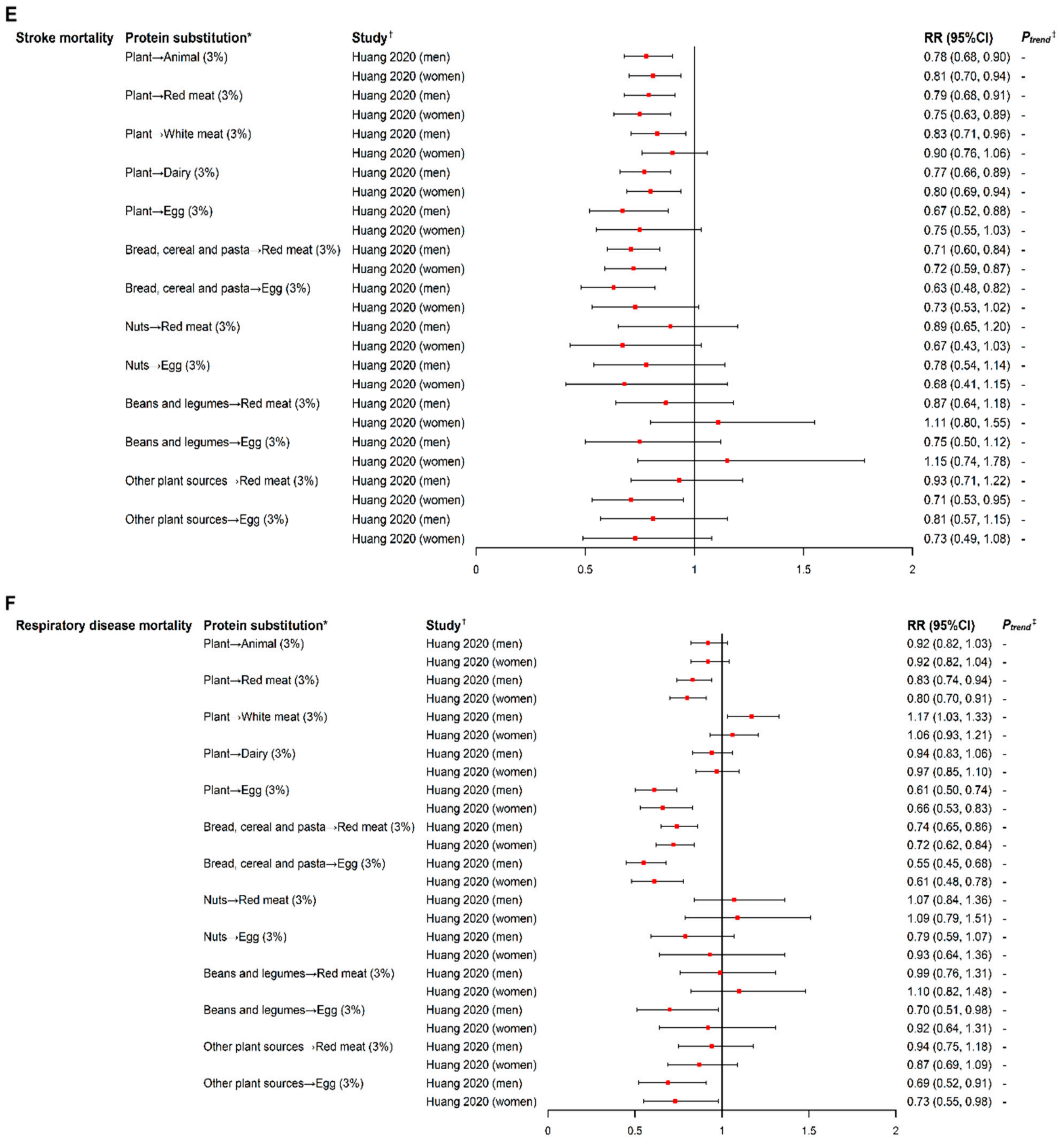

Figure S1. Forest plot of all-cause and cause-specific mortality in relation to isocaloric substitution of plant-based protein for animal-based protein. A) All-cause mortality; B) total cancer mortality; C) cardiovascular disease mortality; D) heart disease mortality; E) stroke mortality; F) respiratory disease mortality. *Symbol "→" represents substitution of protein from the left-side food source for protein from the right-side food source. "Plant" and "Animal" stands for "all the plant-based food sources" and "all the animal-based food sources" respectively. The content in the bracket after the substitution exposure describes the substituted exposure's unit and data type: "Q5 vs Q1" was Quintile 5 versus Quintile 1 of percentage of energy from total plant protein substituted for total animal protein, while "3%" or "5%" referred to substitution of 3% or 5% of total energy intake from various plant-based proteins for various animal-based proteins. †The content in the bracket after reference of publication indicated the population in the analysis, both sexes combined if not otherwise indicated. ‡The P_{trend} value was only reported for the categorical substituted exposure.
